# Individual and community characteristics associated with premature natural and drug-related deaths in 25–59 year old decedents

**DOI:** 10.1371/journal.pone.0212026

**Published:** 2019-02-27

**Authors:** Stacy A. Drake, Yijiong Yang, Dwayne A. Wolf, Thomas Reynolds, Sherhonda Harper, Antoinette Hudson, Janet C. Meininger

**Affiliations:** 1 Department of Research, The University of Texas Health Science Center at Houston, Cizik School of Nursing, Houston, Texas, United States of America; 2 Pathology Department, Harris County Institute of Forensic Sciences, Houston, Texas, United States of America; 3 Department of Management, Policy and Community Health, The University of Texas Health Science Center at Houston, School of Public Health, Houston, Texas, United States of America; 4 Recovery Department, Americares, Stamford, Connecticut, United States of America; 5 Administration, CHI Baylor St. Luke’s Medical Center Houston, Texas, United States of America; 6 Systems Department, The University of Texas Health Science Center at Houston, Cizik School of Nursing, Houston, Texas, United States of America; Sciensano, BELGIUM

## Abstract

The purpose of the study was to identify circumstances of death, disease states, and sociodemographic characteristics associated with premature natural and drug-related deaths among 25–59 year olds. The study also aimed to address the paucity of research on personal, community-based, and societal factors contributing to premature death. A population-based retrospective chart review of medical examiner deaths within a highly populated and ethnically diverse county [in Texas] was undertaken to identify individuals dying prematurely and circumstances surrounding cause of death [in 2013]. The sample data (n = 1282) allowed for analysis of decedent demographic variables as well as community characteristics. Descriptive statistics, multivariable logistic regression, and geospatial analyses were used to test for associations between the type of death (natural or drug-related) and demographics, circumstances of death, disease types and community characteristics. Census tract data were used to determine community characteristics. Highly clustered premature deaths were concentrated in areas with low income and under-educated population characteristics. Two-thirds of decedents whose death were due to disease had not seen a healthcare provider 30 days before death despite recent illness manifestations. Opioids were found in 187 (50.5%) of the drug-related deaths, with 92.5% of deaths by opioids occurring in combination with other substances. The study findings went beyond the cause of death to identify circumstances surrounding death, which present a more comprehensive picture of the decedent disease states and external circumstances. In turn, these findings may influence the initiation of interventions for medically underserved and impoverished communities.

## Introduction

Within the United States (U.S.), premature deaths are rising, driven by both natural diseases and other circumstances, including drug-related causes [1]. Main causes of death are known and reported annually [2]. For the past few decades, multiple studies have identified the importance of characteristics that contribute to mortality other than traditional medical care, e.g., income, education, occupational status, social class [3–11]. Many of these previous studies have been ecological in design, i.e., characteristics of individuals have been related to death rates within the group aggregate. This study provides a different perspective; we describe the characteristics associated with premature deaths of individuals from natural and drug-related causes.

Premature deaths are commonly defined as deaths that occur before an expected mortality age (e.g., 75 years) [1,12]. Research generally focuses on specific causes of premature death (e.g., cardiac disease, cancer, unintentional deaths) [13,14]. However, the current study expands investigations on the causes of premature death including personal and community-based characteristics associated with the cause of death; the disease state, which is defined as the overall picture of the interrelationship of demographics, economic aspects of the geographical locale of death, and societal characteristics; and sociodemographic and community characteristics, which include deaths occurring in medically-underserved areas (MUA).

The medicolegal death investigation (MLDI) system routinely investigates unexpected deaths. The MLDI system takes jurisdiction when decedents do not have a healthcare provider or known medical history, the death occurred at home, the death was unwitnessed, and the circumstances surrounding death suggest the death was non-natural [15]. Accordingly, individuals who die prematurely and unexpectedly from natural causes are within MLDI jurisdiction. Medicolegal death investigations typically include acquiring information related to medical history, scene or home observations, family interviews, and autopsy results (including drug history, inventory, and postmortem analysis). This information provides a database for investigating circumstances surrounding death (CD), disease state (DS), and community characteristics (CC).

To organize the large amount of data available from MLDI records, the research team utilized the Socio-Ecological Model (SEM) [16], which allows for the description of variables influencing premature deaths in terms of individual, interpersonal, organizational, and community characteristics (Table 1). In this study, *individual characteristics* relate to cause and manner of death, circumstances of death, and individual characteristics (such as use of drugs, tobacco, and alcohol); *interpersonal characteristics* relate to status markers, such as unemployment, homelessness, and marital status; *organizational characteristics* refer to access to or documented visit to a healthcare provider; and *community characteristics* are those obtained from the 2013 American Community Survey 5-year data at the block group level [17].

**Table 1:** Theoretical concepts and definitions of study domains and variables.

Model Concepts	Conceptual Definition	Operational Definition	Study Variables	Study Domain
Individual	Individual characteristics that influence behavior.	Decedent characteristics from investigation information used to infer socioeconomic status, documented medical, psychiatric, and/ or social histories.	Age, sex, race, disabled, YPLL^a^, COD^b^ and contributing causes, MOD^c^, BMI^d^, findings of recent illness, documented medical/surgical/ psychiatric/social history, and current alcohol, tobacco, illicit or prescription abuse.	CD^e^ DS^f^
Interpersonal	Formal and informal social networks and social support systems that can impact individual behavior.	Decedent formal and informal social networks.	Lived alone, marital status, homeless, unemployed, decomposed.	CD
Organizational	Organizations or service centered institutions with rules and regulations for operations that affect how, or how well services are provided to an individual.	Investigative information may provide family interviews may indicate access to HCP or recent visit to HCP.	Had a HCP^g^, recent visit to HCP [within 30 days], COD or contributing COD same as antemortem diagnosis.	CC^h^ DS CD
Community	Socioeconomic characteristics of the community within defined boundaries	GIS geomapping will provide details regarding community characteristics and resources.	Home address including zip code with maps to include census track level transportation, education, unemployment rate, median household income, medically underserved areas and GINI co-efficient.	CC

^a^YPLL = Years of Potential Life Lost.

^b^COD = cause of death.

^c^MOD = Manner of Death.

^d^BMI = Body Mass Index.

^e^CD = Circumstances of Death.

^f^DS = Disease States.

^g^HCP = Healthcare Provider.

^h^CC = Community Characteristics.

In 1986, the Centers for Disease Control and Prevention (CDC) used years of potential life lost (YPLL) as a measure to report on premature mortality [18]. The use of YPLL provides a quantitative measure that assigns more weight to deaths occurring unexpectedly at younger ages than at older ages. Therefore, YPLL is included as a study variable at the individual level that falls within the CD and DS domains. Geocoding and mapping are operational aspects within the community level of the SEM that permit visualizing communities where premature deaths are clustered.

Between 2000 and 2015, premature deaths due to drug overdose increased 137%, thus making drug toxicity a chief contributor to premature deaths [11]. For the present study, because comparable data were available for drug-related deaths and for natural deaths, a sample of premature drug-related deaths served as a comparison group. Premature natural death refers to deaths of individuals aged 25–59 years of age due solely to the effects of disease. Premature drug-related death refers to deaths of individuals aged 25–59 years of age attributed to drug toxicity (e.g., prescription drugs, illicit drugs, alcohol, drug combinations). In some instances, drug combinations represented an overdose of prescribed medications; in other instances, these were deaths from non-prescribed (i.e., illicit) substances for which no appropriate “dose” existed.

### Purpose of the study

We conducted a study using MLDI records from a highly populated county in the south central U.S. to identify circumstances of death; disease; and sociodemographic and community characteristics associated with premature natural and drug-related causes of death. Data were organized in terms of individual, interpersonal, organizational, and community characteristics to aid in presentation and interpretation. The aims of the study were: (1) to describe the distribution of premature deaths attributed to natural or drug-related causes by demographics, YPLL, interpersonal variables (e.g., living alone, homelessness) and known use of medical care; (2) to examine the likelihood of death from natural causes compared to drug-related causes in relation to individual, interpersonal, organizational, and community characteristics; and (3) to identify where premature deaths occurred within the study county in order to examine premature deaths in relation to community sociodemographic characteristics.

The study was designed to identify circumstances that contributed to premature death and included personal and community characteristics that might have affected risk of death. Prior literature suggests that community characteristics contribute to the risk of prematurely dying [19]. Therefore, an attempt was made to identify personal and community aspects that may affect risk of premature death.

## Methods and materials

The study was a retrospective, comparative study using both decedent-related and population variables to determine circumstances surrounding the cause of death. Data were retrieved from MLDI charts located at the Harris County Institute of Forensic Sciences, which has death investigation jurisdiction over Harris County and serves the nation’s fourth largest city. The decedent charts included autopsy findings of routine death investigations. We compared two groups, i.e., deaths from natural causes and from drug-related causes, which allowed for comparison of deaths in terms of individual, interpersonal, organizational, and community aspects (Socio-Ecological Model) and identification of the geographic area where premature deaths were clustered.

Approval for the study was obtained from the Harris County Institute of Forensic Science Center Medical Examiner office and The University of Texas Health Science Center at Houston Institutional Review Board. Variables were extracted, coded, and deidentified. Identifiers were coded only for data analysis purposes.

### Sample and setting

**Fig 1:**
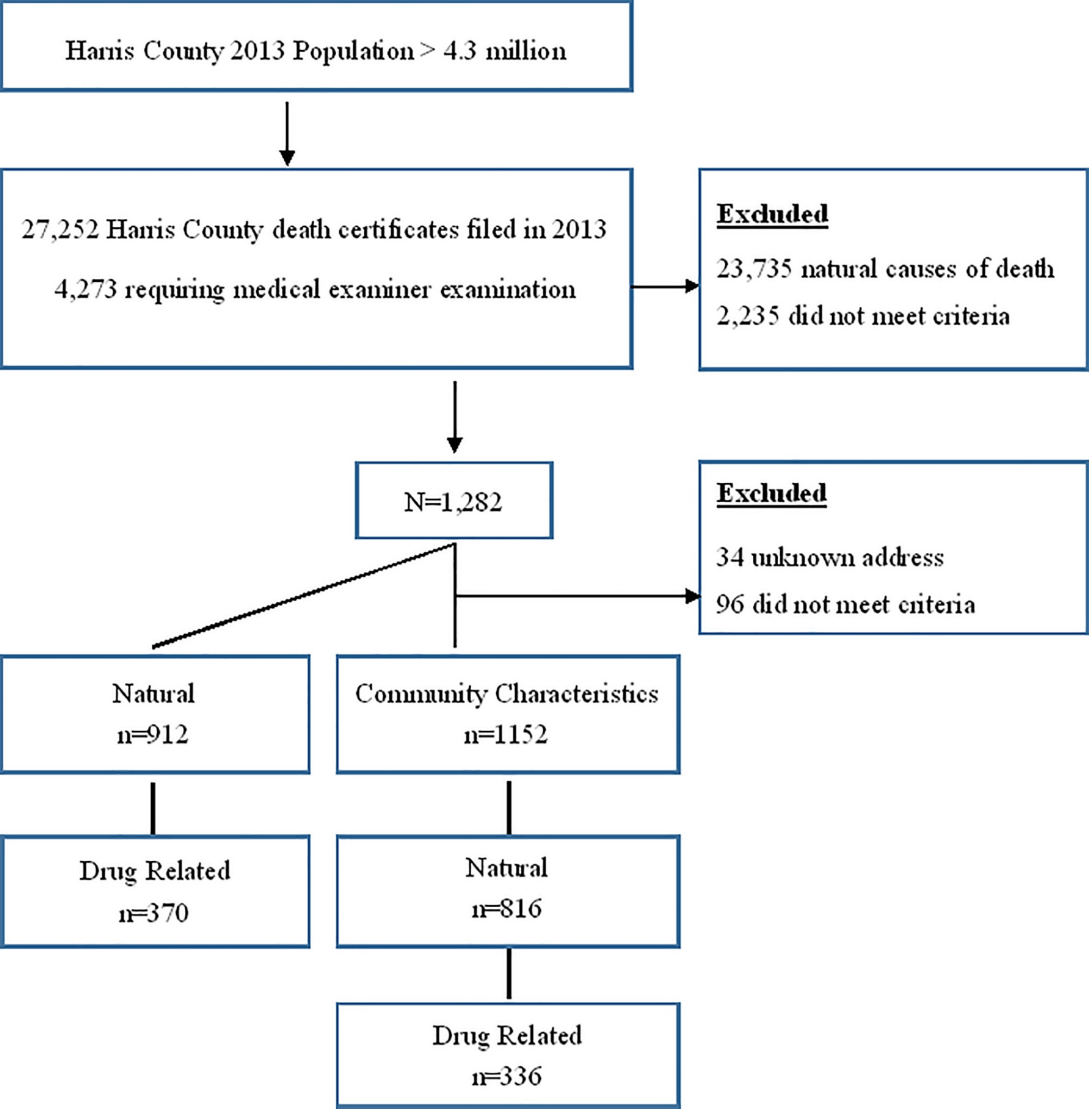
Flow diagram of death cases.

The sample was comprised of 1,282 adults, 25 to 59 years of age, who died from natural causes (natural deaths, n = 912) or drug-related causes (drug-related deaths, n = 370) within a largely populated and ethnically diverse county in calendar year 2013 (Fig 1). The deaths reviewed were within the authority of the MLDI.

We focused on individuals aged 25–59 years of age in an effort to eliminate the youngest and oldest decedents, because deaths in the younger population are more frequently due to traumatic accident or homicide while deaths in the older population are often from known or expected causes [20]. In 2013, MLDI records included 1,668 deaths from natural causes, of which 5.6% (n = 93) occurred in individuals under 25 years of age and 39.7% (n = 663) occurred in individuals over 59 years of age. Thus, the remaining 54.7% (n = 912) were within the age range of this study (25–59 years). Of the 523 deaths identified as drug-related deaths, 15.6% (n = 82) occurred in those less than 25 years of age and 13.5% (n = 71) occurred among those over 59 years of age. The remaining 70.8% (n = 370) met the age criterion.

The setting was Harris County, an area of almost 2,000 square miles with an ethnically diverse population of over 4 million residents. The largest city, Houston, had a population greater than 2 million people located over 600 square miles. According to 2016 U.S. Census population estimates for Harris County, the estimated county-level racial/ethnic composition was 42.4% Hispanic, 30.4% White, 19.7% Black, and 7.2% Asian [21].

### Data collection

The investigators developed a standardized procedure for retrieving data from MLDI records. The form for data retrieval contained 47 items relating to decedent demographic and community characteristics, antemortem health history, circumstances surrounding death, and postmortem findings. Responses were recorded as Yes, No, or Unknown (e.g., “Lived alone?”), direct data input (e.g., date of birth), or retrieved information (e.g., cause of death). For content validity purposes, four forensic experts reviewed the form. The codes used for both cause of death and underlying cause of death were the 2013 International Classification of Diseases Codes (ICD-10) [22]. Data for the community characteristics domain were ascertained from 2009–2013 American Community Survey (ACS) 5-year census tract data [17]. Characteristics of the population at the census tract or block group level pertained to those aged 25–59 whenever possible.

### Procedure

Forensic nurse specialists and undergraduate student nurses trained to use the data retrieval tool abstracted data from decedent’s records on-site at the Medical Examiner’s office. The training consisted of a step-by-step abstraction of actual cases, followed by independent abstraction of two deaths: one natural and one drug-related. A team of five individuals, including the PI, met weekly to abstract cases. For every 100 cases, 10% of the records were randomly selected for assessment of inter-rater reliability (IRR) of coding. The IRR for use of the tool was 90%. Subsequent IRRs per 10 of 100 cases ranged from 70% to 100%. When the IRR of coding fell below 90%, data were recoded and retraining occurred.

### Data management and analyses

#### Premature death data

Data were recorded and managed using an electronic data capture system, *REDCap* (Research Electronic Data Capture), hosted by the PI’s university. Statistical analyses were conducted using IBM SPSS Statistics (version 22.0). Descriptive statistics presented an overview of sample characteristics (e.g., demographics, circumstances of death, disease states) and chi-square or Kruskal-Wallis tests were used to assess significance. The variables for height; weight; body mass index (BMI); pathology; cause and contributing cause of death; and manner of death were derived from autopsy reports, while other items were based on details from scene investigations and recorded interviews with next of kin or others. The YPLL was calculated based upon 2013 life expectancy by sex, with females at 81.2 years of age and males at 76.4 years of age [23]. Cause and contributing causes of death were categorized based upon ICD-10 indexes.

Multivariable logistic regression analysis was performed to differentiate those characteristics significantly associated with drug-related deaths compared with natural deaths; odds ratio, 95% confidence interval, and P-value were calculated. When using G*Power to calculate the effect size of two-tailed odds ratio with sample size of 1,282 and categorical predictors with a 0.80 power at the 0.05 alpha level, the significant odds ratio should be lower than 0.69 or higher than 1.44. Independent variables included the individual, interpersonal, and organizational characteristics listed in Table 1. Variables with unknown values exceeding 20% (e.g., unemployment status) or those with no variability (100% or 0% in one of the groups, e.g., disabled in the drug related group) were excluded from analysis. Variables for the model were selected by backward elimination with p < .05 for entry and p ≥ .10 for removal. First, all independent variables were entered, then the nonsignificant variables were eliminated one at a time in order to retain those that were significant while controlling for all other variables in the model. In the final model, odd ratios with 95% confidence intervals were adjusted for other variables in the model [24].

#### Community-based data

Community area designations of geographic locale of both natural deaths and drug-related deaths were geocoded based on the address of where decedents were found (place of death in the case of scene deaths, or place where transported from in the case of hospital deaths) as well as their reported residential address. To accomplish population mapping, the home or resident addresses were geocoded to x and y coordinates (longitude and latitude) using three locator files and ArcMap 10.5.1. Addresses that could not be geocoded after the initial runs were manually corrected. The majority of errors were due to misspelling of street names and improper entries in the variable fields. Cases with less than 85% accuracy were removed from the analytical data set.

The original premature death data set contained 1,282 cases (Fig 1). For the geospatial analysis, 34 cases were removed due to incomplete or unknown addresses. This resulted in 1,248 cases available for analysis. As part of the initial geospatial analysis, 96 more cases were removed from the analytical data set because residential addresses were outside the set county boundaries. Of the remaining 1,152 cases, 816 were natural deaths and 336 were drug-related (Fig 1). Both the single and dual kernel density analyses were conducted using CrimeStat 4.02 [25, 26]. Spatial distribution of premature deaths was accomplished using both single and dual kernel density estimation to generalize incident locations for an entire area and describe clusters in relation to an underlying at-risk [27].

In this study, the case file of premature deaths is the primary file for the single kernel density estimate and serves as the numerator for the dual kernel density interpolation. The secondary file, or denominator file, was developed from the U.S. Census Bureau’s 2009–2013 5-year American Community Survey (ACS) data file [17]. The total population count of ages 25 to 59 at the block group level of geography was the denominator value. The centroid of each block group, designated by longitude and latitude, was used to provide the location of each point for the denominator file. Tracts used to develop community characteristics were selected if they fell completely or partially within the high-risk areas. Percentages were developed from the summed counts. Median household income was determined using a proportional estimation method based on number of households in each census tract. A Gini coefficient (an index of income inequality) was calculated, but because the Gini coefficient is computed from reported household income, it cannot be disaggregated by age. The Gini index ranges from 0.0 to 1.0, with 0.0 indicative of perfect equality and 1.0 indicative of perfect inequality [25].

## Results

The sample data set was comprised of 1,282 premature deaths from natural (n = 912) or drug-related (n = 370) causes. The individual-related characteristics studied are presented in the model depiction (Table 1).

### Sample characteristics

Sample descriptive statistics for the natural and drug-related deaths are shown in Table 2. Also presented are statistical differences between the two groups.

**Table 2:** Decedent characteristics overall and for premature natural and drug-related deaths (n = 1282).

Variable	All Groups n (%) of Total 1,282 (100%)^a^	Natural n (%) 912 (71.1%)	Drug Related n (%) 370 (28.9%)	P value Significance of differences between groups
**Individual Concept**				
Age in years (mean ±SD)	47.3 (9.1)	48.5 (8.5)	44.2 (9.8)	< .001
Sex (male) (n [%])	865 (67.5)	643 (70.5)	222 (60.0)	< .001
Racial Group^b^ (n [%])				< .001
White	661 (51.6)	430 (47.1)	231 (62.4)	
Black	355 (27.7)	284 (31.1)	71 (19.2)	
Hispanic	232 (18.1)	174 (19.1)	58 (15.7)	
Asian	34 (2.7)	24 (2.6)	10 (2.7)	
Disabled	1,035 (80.7)	665 (72.9)	370 (100)	< .001
Years of Potential Life Lost (YPLL)				
Male (mean ±SD)	28.9 (8.96)	27.5 (8.22)	32.8 (9.88)	< .001
Female (mean ±SD)	34.5 (9.40)	33.6 (9.19)	36.1 (9.58)	< .01
BMI mean (±SD)	29.9 (9.2)	30.1 (9.6)	29.5 (8.1)	.473
Recent illness^c^ (n [%])	693 (54.1)	533 (58.4)	160 (43.2)	< .001
Documented past medical, psychological, surgical or social history (n [%])	1,053 (82.1)	744 (81.6)	309 (83.5)	.049
Current alcohol use^d^	694 (54.1)	441 (48.4)	253 (68.4)	< .001
Current tobacco use^d^	601 (46.9)	384 (42.1)	217 (58.6)	< .001
Current substance use^d^	522 (40.7)	196 (21.5)	326 (88.1)	< .001
**Interpersonal Concept**				
Marital Status (n [%])				.353
Single	876 (68.3)	613 (67.2)	263 (71.1)	
Married	380 (29.6)	281 (30.8)	99 (26.8)	
Unknown	26 (2.0)	18 (2.0)	8 (2.2)	
Lived Alone	408 (31.8)	310 (34.0)	98 (26.5)	< .001
Homeless	82 (6.4)	48 (5.3)	34 (9.2)	.009
Unemployed	416 (32.4)	282 (30.9)	134 (36.2)	< .001
Found in decomposed condition	160 (12.5)	120 (13.2)	40 (10.8)	.249
**Organizational Concept**				
Had a healthcare provider^e^ (n [%])	627 (48.9)	439 (48.1)	188 (50.8)	.020
Visited a healthcare provider within last 30 days (n [%])	377 (29.4)	275 (30.2)	102 (27.6)	< .001
Cause or contributing cause of death the same as antemortem diagnosis^f^ (n [%])	950 (74.1)	637 (69.8)	313 (84.6)	< .001

^a^Totals for some variables are less than 1,282 due to unknown values.

^b^Mutually exclusive categories.

^c^Included scene findings or interview data suggestive of acute illness within 30 days of death.

^d^Current was defined as documented, stated or scene findings of use at time of death.

^e^Included as a stated or documented presence of healthcare provider at time of death.

^f^Cause or contributing cause of death found on the death certificate was the same as antemortem diagnosis.

#### Individual characteristics

Of the total sample (n = 1,282), 51.6% (n = 661) was white and 67.5% was male (n = 865). The mean age of natural deaths was higher (u = 48.5, SD = 8.5) than the mean age for drug-related deaths (u = 44.2, SD = 9.8). For natural deaths (n = 912), the YPLL ranged from 17.4 years to 56.2 years, with a mean of 29.3 years (SD = 8.94). For drug-related deaths (n = 370), the YPLL was higher than for natural deaths, with a mean of 34.1 years (SD = 9.88). In a subset analysis of YPLL, the mean (33.6 years, SD = 9.19) of females of natural death (n = 269) was lower than the mean (36.1 years, SD = 9.58) of females from drug-related deaths (n = 148). Similarly, the mean YPLL (27.5 years, SD = 8.22) of males of natural death was lower than the mean (32.8 years, SD = 9.86) of males of drug-related deaths. In comparison to natural deaths, a larger proportion of individuals from drug-related deaths reported current alcohol usage (68.4%, n = 253 versus 48.4%, n = 441), current tobacco usage (58.6%, n = 217 versus 42.1%, n = 384) and current substance usage (88.1%, n = 326 versus 21.5%, n = 196). Of the 912 natural deaths, 58.4% (n = 533) had a history of recent illness.

#### Interpersonal characteristics

Of the total sample, 68.3%, (n = 876) were single. In comparison with the natural deaths, a higher number of individuals from drug-related deaths (30.9%, n = 282 versus 36.2%, n = 134) were unemployed. In comparison with the natural deaths, a lower number of individuals from drug-related deaths (10.8%, n = 40 versus 13.2%, n = 120) were found in a decomposed condition.

#### Organizational characteristics

Of the total sample, 48.9% (n = 627) had a healthcare provider; a larger proportion of drug-related deaths compared with those with natural deaths (50.8%, n = 188 versus 48.1%, n = 439) had a healthcare provider. In the total sample, (29.4%, n = 377) had visited a healthcare provider within the last 30 days before death. In comparison with the natural deaths, a lower number of individuals from drug-related deaths (27.6%, n = 102 versus 30.2%, n = 275) had visited a healthcare provider within the last 30 days.

#### Variables associated with drug-related versus premature natural deaths

Results of multivariable logistic regression with cause of premature death (drug-related versus natural) as the dependent variables are presented in Table 3 (R-square = 0.546). Higher value of years of potential life lost was associated with higher odds of drug-related death (OR = 1.04, 95% CI = 1.02–1.06, P value < .001) while controlling for all other variables in the model. Blacks had lower odds of drug-related death than whites (OR = 0.45, 95% CI = 0.30–0.67, P value < .001); married people had higher odds than single (OR = 1.77, 95% CI = 1.16–2.70, P value = .01). With regard to alcohol and substance use, current alcohol use increased the odds of drug-related death (OR = 1.82, 95% CI = 1.25–2.64, P value = .002), and odds were significantly higher for those with current substance use compared without current substance use (OR = 25.10, 95% CI = 16.65–37.85, P value < .001). Odds of drug-related deaths were lower for individuals with a history of recent illness (OR = 0.50, 95% CI = 0.35–0.74, P value < .001). The odds of drug-related death compared with natural death were higher for those with cause or contributing cause of death the same as antemortem diagnosis (OR = 1.99, 95% CI = 1.31–3.03, P value = .001). The overall significance of Hosmer-Lemeshow goodness of fit of the final model was 0.814, with classification rate 83.1% (premature natural death predicted correctness 87.5%, drug related death predicted correctness 72.2%) [28].

**Table 3:** Multivariable logistic regression analysis of drug-related versus premature natural deaths (n = 1,282).

	Drug-related versus Premature Natural Deaths			
	Odds Ratio	95% CI	Wald Test Value	P Value
**Years of Potential Life Lost**				
YPLL	1.04	1.02–1.06	20.56	< .001
**Race**				
White	1	[Reference]		
Black	0.45	0.30–0.67	15.20	< .001
Hispanic	0.76	0.48–1.23	1.23	0.27
Asian	1.03	0.34–3.09	0.003	0.96
**Marital Status**				
Single	1	[Reference]		
Married	1.77	1.16–2.70	6.94	0.01
Unknown	1.19	0.35–4.03	0.07	0.79
**Current Alcohol Use**				
Yes	1.82	1.25–2.64	9.82	0.002
No	1	[Reference]		
**Current Substance Use**				
Yes	25.10	16.65–37.85	236.73	< .001
No	1	[Reference]		
**Lived Alone**				
Yes	0.80	0.54–1.20	1.17	0.28
No	1	[Reference]		
**Recent Illness**				
Yes	0.50	0.35–0.74	12.534	< .001
No	1	[Reference]		
**Visited Healthcare Provider**				
Yes	0.97	0.65–1.45	0.03	0.88
No	1	[Reference]		
**Cause or Contributing Cause of Death the Same as Antemortem Diagnosis**				
Yes	1.99	1.31–3.03	10.45	0.001
No	1	[Reference]		

### Cause of death

#### Natural deaths

In relation to the primary causes of death, diseases of the circulatory system were the most frequent cause of premature natural deaths (62.3%, n = 568). The next most common causes were diseases of the digestive system (6.9%, n = 63); endocrine, metabolic and nutritional diseases (6.6%, n = 57); and diseases of the respiratory system (5.7%, n = 52). Of the 912 natural deaths, 36% (n = 324) included a contributing cause of death. Diseases of endocrine, nutritional and metabolic diseases, including obesity, were the most frequent (57.4%, n = 186). The next most common contributing causes were diseases of circulatory system (12.3%, n = 40), respiratory (12.0%, n = 39), and mental behavioral disorder, including chronic substance abuse (7.1%, n = 23). Additionally, these three causes (circulatory, respiratory, mental behavior disorder) occurred in various combinations (4.6%, n = 15). In terms of recent illness manifestation, 51.1% (n = 466) were classified as general symptoms, such as fatigue and headache, although for 30.9% (n = 281), recent illness manifestations were unknown. Recent illness manifestations for the remaining deaths were as follows: gastrointestinal or digestive, 10.7% (n = 98); cardiac, 5.2% (n = 47); musculoskeletal, 1.1% (n = 10); and neurological, 1.0% (n = 9).

The association between cause of death and recent illness manifestations was significant (p < .001). Circulatory deaths were associated with general and cardiac illness manifestations. Digestive as well as endocrine causes of death were associated with gastrointestinal or digestive manifestations. Respiratory system causes of death tended to have general or unknown illness manifestations.

#### Drug-related deaths

Of the drug-related deaths, 90.5% (n = 335) were classified as accidental and 9.5% (n = 35) as suicide. The substance descriptions for drug-related causes of death are shown in Table 4, which was organized not only by the type of drug (illicit, prescription, alcohol or over-the-counter) but also by the specific drugs within each category type.

**Table 4:** Substance description for drug-related premature deaths (n = 370).

Variable	A single substance as primary cause of death^a^ n (%)	Combination of substances listed as cause of death^b^ n (%)	Total Observations^c^ n
**Illicit**			
*Cocaine*	105 (63.6)	60 (36.4)	165
*Heroin*	8 (22.9)	27 (77.1)	35
*Methamphetamine*	23 (76.7)	7 (23.3)	30
*Phencyclidine*	12 (70.6)	5 (29.4)	17
*Designer*	1 (12.5)	7 (87.5)	8
**Prescription**^d^			
Opioid	14 (7.5)	173 (92.5)	187
*Hydrocodone*	5 (5.0)	96 (95.0)	101
*Morphine*	2 (8.3)	22 (91.7)	24
*Oxycodone*	0 (0)	20 (100.0)	20
*Fentanyl*	5 (35.7)	9 (64.3)	14
*Other*^e^	2 (7.1)	26 (92.9)	28
Muscle Relaxant			
*Carisoprosal*	0 (0)	29 (100.0)	29
Benzodiazepines	0 (0)	122 (100.0)	122
*Alprazolam*	0 (0)	50 (100.0)	50
*Diazepam*	0 (0)	38 (100.0)	38
*Zolpidem*	0 (0)	17 (100.0)	17
*Clonazepam*	0 (0)	12 (100.0)	12
*Other*^f^	0 (0)	5 (100.0)	5
Antidepressants	6 (5.6)	101 (94.4)	107
Antipsychotics			
*Trazadone*	0 (0)	20 (100.0)	20
*Bupropion*	0 (0)	18 (100.0)	18
*Quetiapine*	1 (5.6)	17 (94.4)	18
*Citalopram*	1 (6.3)	15 (93.7)	16
*Other*^g^	4 (11.4)	31 (88.6)	35
Anticonvulsant			
*Gabapentin*	0 (0)	9 (100.0)	9
Antihistamines^h^	1 (1.9)	53 (98.1)	54
Other^i^	5 (45.5)	6 (54.5)	11
**Alcohol**^**j**^	20 (19.4)	83 (80.6)	103
**Over the Counter**			
*Acetaminophen*	2 (9.5)	19 (90.5)	21
*Other*^*k*^	0 (0)	9 (100.0)	9

^a^Forensic toxicology results revealed a single substance as the cause of death.

^b^Forensic toxicology results revealed a combination of substances to include alcohol, illicit, prescription(s) or over the counter.

^c^Total number of observations identified from toxicology results.

^d^Does not infer an actual prescription was provided for obtaining the substance.

^e^Includes tramadol, codeine, methadone, oxymorphone and hydomorphone.

^f^Includes temazepam and chlordiazepoxide.

^g^Includes milnacipram, amitriptyline, mirtazapine, venlafaxine, fluoxetine, paroxetine, doxepin, sertraline, olanzapine and risperidone.

^h^Includes both prescription antihistamine(s) (promethazine, chlorpheniramine) and over-the counter (doxylamine, diphenhydramine).

^i^Includes difluoraethane, lidocaine, topiramate, insulin, propranolol and unspecified barbiturate.

^j^Acute toxicity only.

^k^Includes guaifenesin, salicylate, naproxen and dextromethorphan.

According to toxicology results, cocaine accounted for the highest number of deaths due to a single substance (28.4%, n = 105). Opioids were found in 187 (50.5%) of the deaths, with 92.5% of deaths by opioids occurring in combination with other substances. Although benzodiazepines were not found as a single cause of death, this class of drugs was found in combination with other substances (33%, n = 122), as were antidepressants (27.3%, n = 101) and antipsychotics (27.3%, n = 101). Both prescription strength and over-the-counter antihistamines were found in combination with other substances (14.3%, n = 53). Acute alcohol toxicity accounted for 20 deaths (5.4%), and alcohol was found in an additional 83 (22.4%) deaths combined with other substances. Over-the-counter medications were observed at toxic levels in 30 (8.1%) deaths.

### Geospatial analysis and community characteristics

The dataset, after removal for incomplete data, unknown values, or out-of-area addresses, contained 1,152 deaths for geospatial analysis. Natural deaths were 70.8% of all deaths (n = 816) and drug-related deaths were 29.2% of all deaths (n = 336).

**Fig 2:**
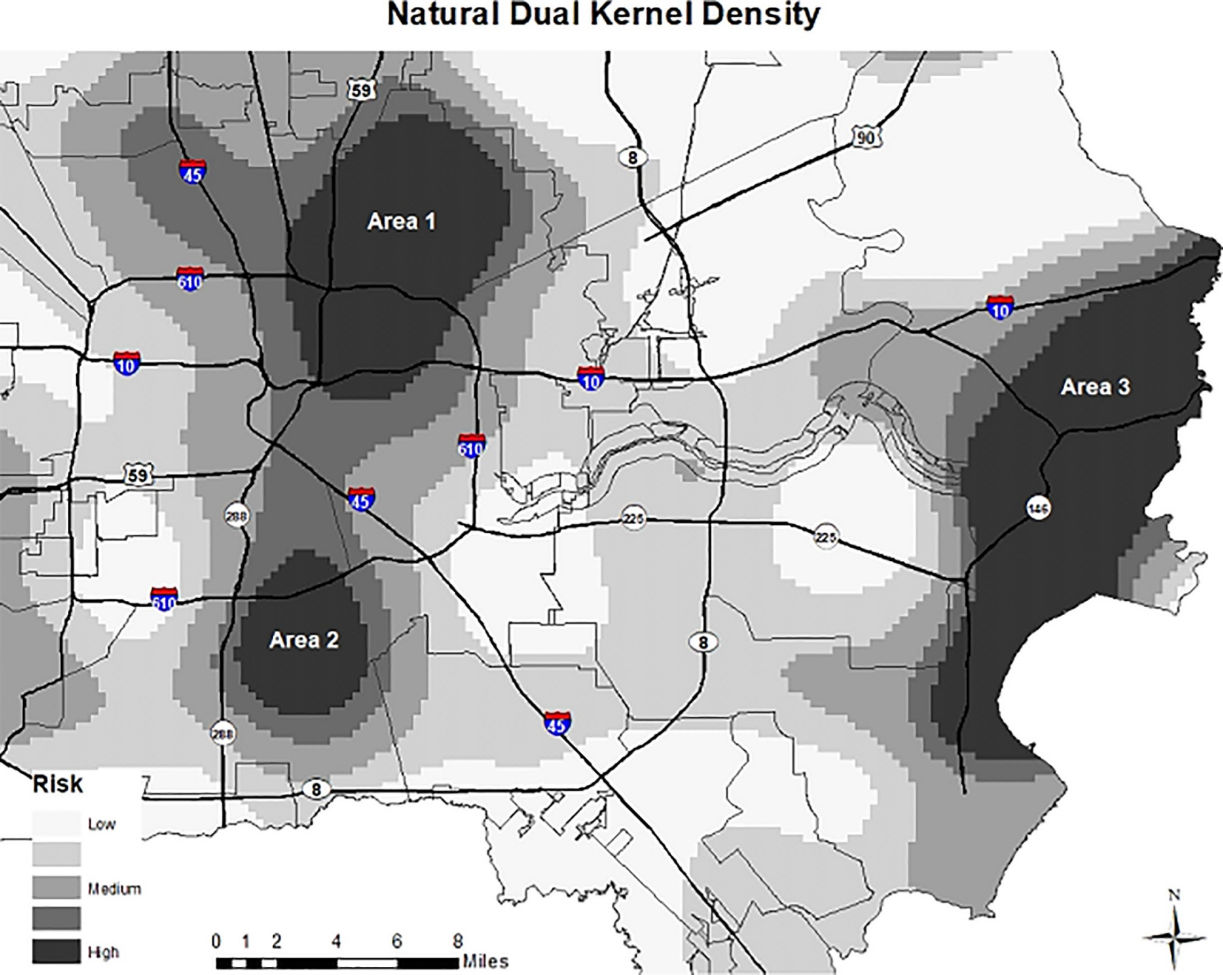
Duel kernel density estimation for premature natural deaths.

The results of the dual kernel density estimation for the natural deaths are shown in Fig 2. This map shows the ratio of densities, where the numerators are the incident events (set of point locations) of premature natural deaths and the denominator is the block-group age-associated population. The three areas highlighted in darkest gray are where the risk for premature natural deaths is highest. Two urban areas central to the city are highlighted. An area of concentration in the eastern part of the county includes a heavily industrial city with a population approximately 72,000. Although natural deaths clustered in 3 distinct areas, the denominator of where they clustered varied; for the numerators, Area 1 (North Central), had 52 natural death events, Area 2 (South) had 29 events, and Area 3 (East) had 34 events. In total, the 115 deaths included in these clusters represent 14.1% of the natural deaths in the sample.

Selected community characteristics of the three areas of premature natural death clusters are presented in Table 5. A difference in neighborhood characteristics among the three areas was that Areas 1 (North Central) and 2 (South) had a higher minority population (> 90%) than Area 3 (East) (59%). In relation to state and local averages, all three areas had a larger population without a high school diploma or equivalent, with Area 1 having the highest (39% vs. state average of 18%); higher unemployment rate, with Area 2 having the highest (19% versus state average of 8%); lower median household income, with Area 1 having the lowest ($28,000 versus state average of $52,000); and a greater percent of people in poverty, with Area 1 having the highest (35% versus state average of 17.5%).

**Table 5:** Census data community characteristics for premature natural deaths.

Area	North Central Area 1	South Area 2	East Area 3	Texas	Harris County	City of Houston
Characteristic						
Percent Minority Population^a^	96.2%	96.6%	59.19%	57.4%	69.6%	74.9%
Percent Black Not Hispanic	47.7%	77.1%	13.82%	12.6%	19.7%	22.4%
Percent Hispanic	48.5%	19.5%	45.37%	39.1%	42.4%	44.3%
GINI Coefficient	0.4354	0.4454	0.4315	0.4751	0.4943	0.5221
No HS Diploma, ≥Age 25	39.3%	26.5%	25.1%	18.1%	21.3%	24.6%
Unemployment Rate	16.6%	18.9%	12.8%	8.1%	8.6%	9.3%
Median Household Income	$28,109	$29,194	$46,694	$51,900	$53,137	$45,010
Percent in Poverty	34.9%	31.7%	22.0%	17.5%	18.5%	22.9%
Percent of Households with No Vehicle	17.5%	20.2%	7.7%	5.9%	7.0%	9.9%

^a^Minority population defined as non-White.

**Fig 3:**
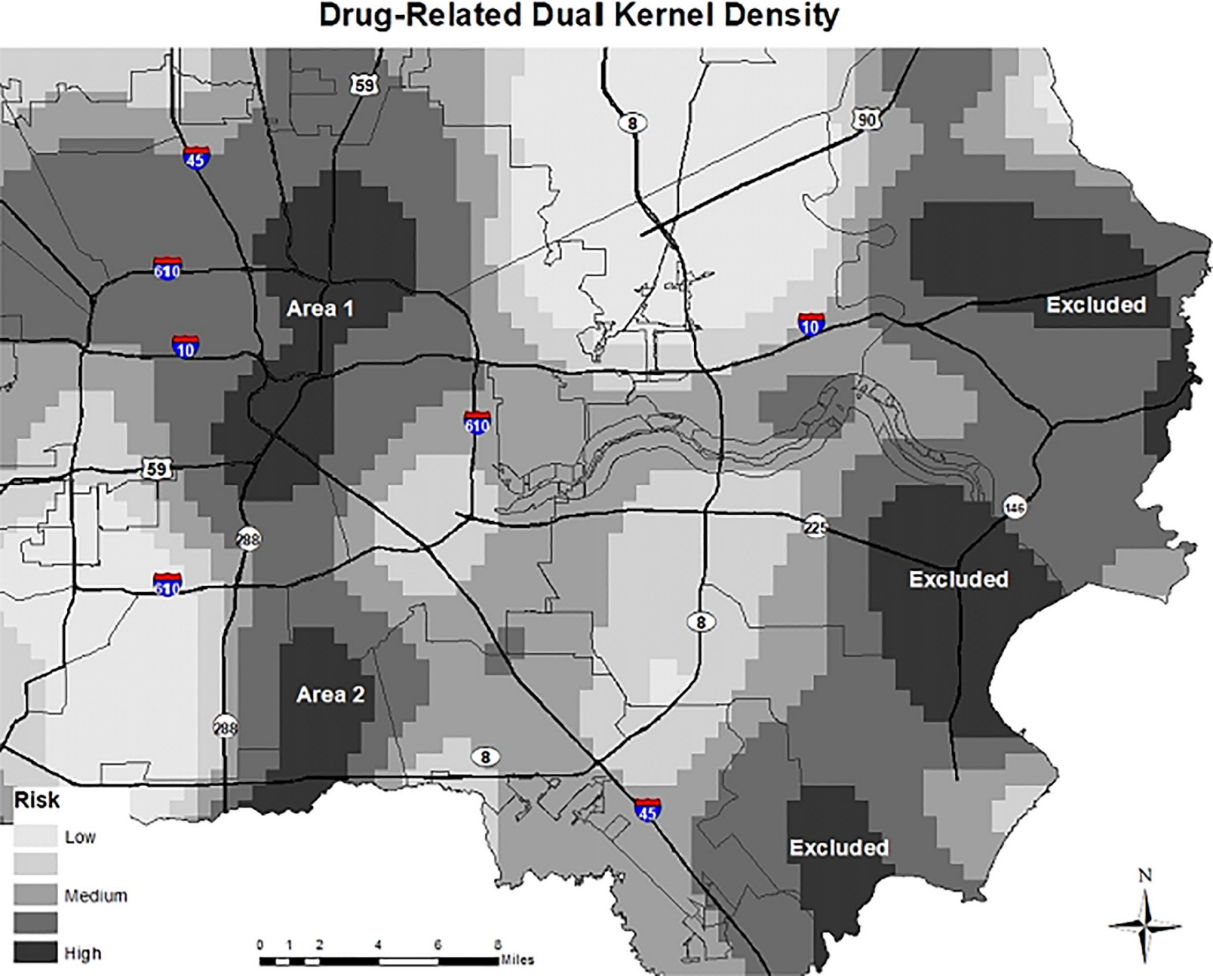
Dual kernel density estimation for premature drug-related deaths.

The drug-related dual density map is shown in Fig 3. Whereas drug-related cases clustered in two distinct areas, the denominators of the clustering varied. The numerators were Area 1 with 40 drug-related events and Area 2 with 13 events. Three areas were excluded because they had fewer than five cases. In total, the 53 events in the two areas included clusters representing 15.8% of the drug-related deaths.

Selected community characteristics of the two areas with clustering of drug-related deaths are presented in Table 6. The percentage of minority population was lower for Area 1 (North Central) (72.4%) than for Area 2 (South) (93.9%), and both areas were higher in minority population than the state average (57.4%). The education level (percent without high school diploma or equivalent) was lower for area 2 (21.5% versus 29.3%) and levels for both areas were higher than the state average (18.1%).

**Table 6:** Census data community characteristics for premature drug-related deaths.

Area Characteristic	North Central Area 1	South Area 2	Texas	Harris County	City of Houston
Percent Minority Population^a^	72.4%	93.9%	57.4%	69.6%	74.9%
Percent Black Not Hispanic	29.2%	75.5%	12.6%	19.7%	22.4%
Percent Hispanic	43.2%	18.4%	39.1%	42.4%	44.3%
GINI Coefficient	0.4762	0.4221	0.4751	0.4943	0.5221
No HS Diploma, ≥ Age 25	29.3%	21.5%	18.1%	21.3%	24.6%
Unemployment Rate	11.3%	17.6%	8.1%	8.6%	9.3%
Median Household Income	$46,465	$38,980	$51,900	$53,137	$45,010
Percent in Poverty	30.3%	23.6%	17.5%	18.5%	22.9%
Percent of Households with No Vehicle	16.2%	13.3%	5.9%	7.0%	9.9%

^a^Minority population defined as non-White.

In comparison with natural deaths, the median household income was higher for clusters of drug-related deaths, and unemployment rate was lower. The highest poverty rate (34.9% versus state average of 18.5%) across the drug-related and natural deaths was for Area 1 for clusters of natural deaths. In terms of Gini coefficients, income inequality was highest for the drug-related group in Area 1.

**Fig 4:**
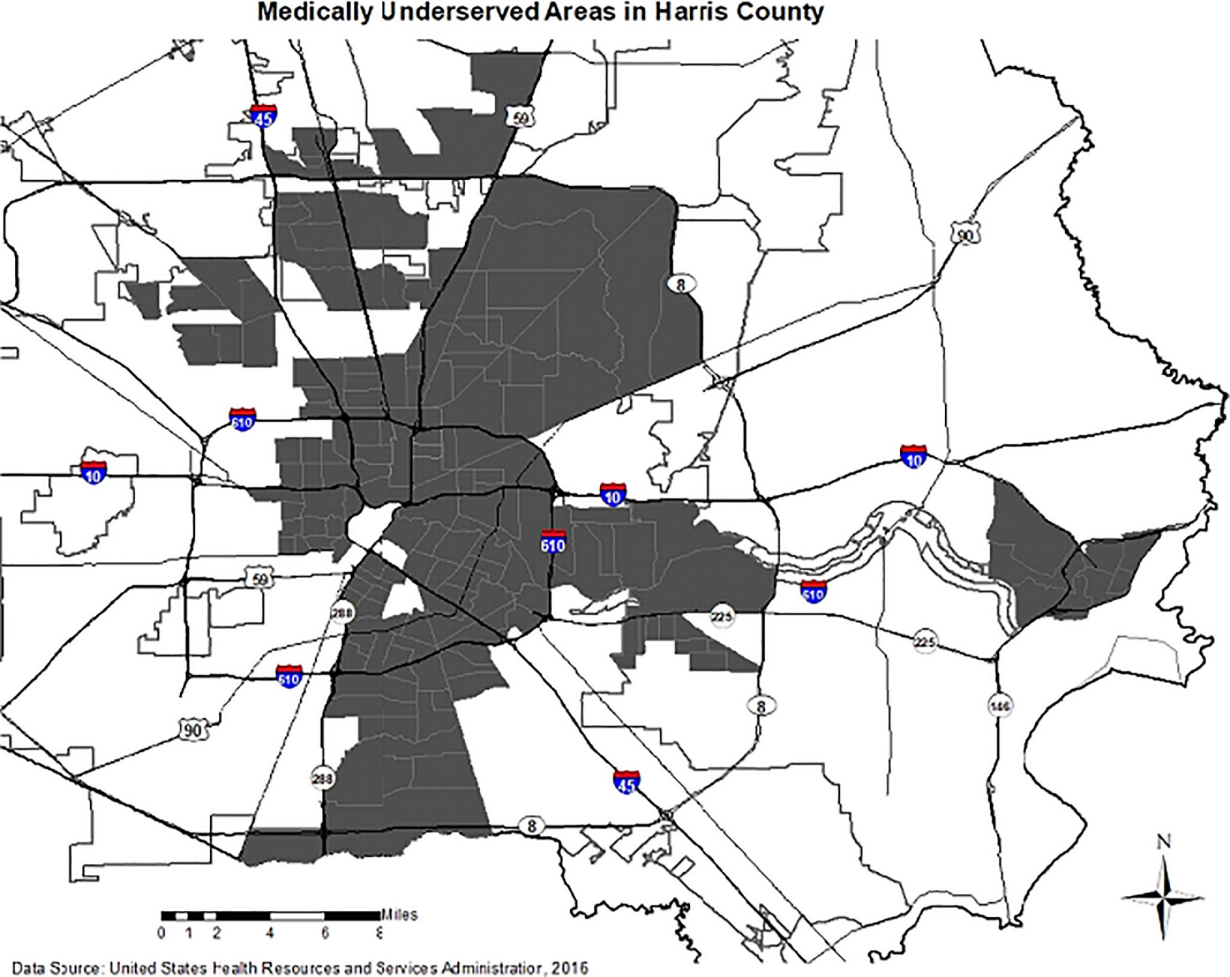
Medically underserved areas.

To gain a preliminary picture of where premature natural deaths clustered in relation to medically-underserved areas, a third analysis was conducted. Based on the dual kernel density results for the premature natural deaths, 53 of the 66 Census tracts that comprise high clusters are designated as MUAs, as shown in Fig 4. Area 1 (North Central) is associated with 28 Census tracts, and all 28 tracts are MUAs. For Area 2 (South), 18 of the 19 tracts that comprise this cluster are MUAs. For Area 3 (East), seven of the 19 tracts comprising it are MUAs.

## Discussion

To our knowledge, this is the first study to investigate local level differences in two growing trends of premature deaths, that of natural pathology and drug-related causes [29, 30]. Although several previous studies have highlighted the growing trend of increasing “midlife” mortality [14, 31–32], these reports capture mainly death certificate information and fail to address detailed circumstances. Other studies provided geographical distributions of drug-related and cause specific mortality over large areas; however, these reports do not provide specific individual, interpersonal, organizational, or community level details [33, 34]. In contrast, the current study presents analysis of detailed death investigation data for individual premature deaths that may translate into data-driven policy and technology interventions to reduce premature deaths at community levels.

In terms of drug-related versus natural deaths, whites had a higher percentage of drug-related deaths, the age group was slightly younger, substance abuse of alcohol and tobacco were higher, and individuals were less likely to have seen a healthcare provider within 30 days of death. In addition, community population socio-demographics were similar for both groups and were characterized by low income and under-education as well as relatively high Gini coefficients (at midpoint between 0 and 1), indicative of relative income inequality. These findings support previous studies regarding income inequality and increased mortality [3, 35–37]. Approximately half of decedents did not have a known healthcare provider and less than a third of decedents sought medical help within 30 days of dying. This suggests that either there were limited healthcare resources available or the individuals, when living, did not comprehend or comply with medical directives or did not have the resources to do so. This lends credence to the premise that there are wide differences in the burden of disease, especially on the state level as shown in a seminal 2018 study [38–39]. In other words, life expectancy could depend largely on where you live [40].

Having expanded knowledge of individual, interpersonal, organizational, and community-based aspects of circumstances surrounding the cause of death provides information toward devising strategies to reduce premature mortality. Efforts to make population-based information available should take priority if effective policies are to be developed toward achieving desirable outcomes.

### Premature natural deaths

Some 62.3% of the premature natural deaths were attributed to diseases of the circulatory system (e.g. atherosclerosis, hypertension, strokes, abdominal aortic aneurisms, heart attacks). These are similar findings to causes of natural death at the U.S. national level [13]. These individuals may have had long term and cumulative exposure to risk factors or contributory lifestyles habits, whether due to socioeconomic and cultural aspects or personal choice, that combined with lack of medical access, potentially contributed to premature mortality [3–10].

However, regardless of the disease state, the disease itself had manifestations. Were these manifestations being ignored, simply accepted, or misunderstood? Two thirds of the individuals did not seek medical help within 30 days of dying, which brings up the question of what was different, or common, between those who did and did not seek help. From the data abstracted from MLDI files, it appears that those with recent illness within 30 days before death experienced symptoms commonly aligned with their antemortem diagnosis. Even so, medical help often was not sought. These results align with a recent study that found middle aged adults often had chest pain and dyspnea up to 4 weeks preceding a sudden cardiac arrest [41].

Of those who had manifestations and visited a healthcare provider (30.2%) within 30 days of death, questions are raised regarding the need for deeper assessment as to the possibility of misdiagnosis or worsening of disease that required new interventions. A report in 2015 addressed the urgency of decreasing missed diagnosis as a priority for improving patient outcomes [42]. Additionally, it is unknown if other social behavioral aspects contributed to overlooking the worsening of disease, e.g., poverty and lifestyle habits of tobacco use, unhealthy diet, lack of exercise coupled with undereducation [7, 31–32].

Of additional clinical interest, social isolation may be an influencing factor in individuals not seeking healthcare before symptoms worsen. [43] Two-thirds of the decedents were single, and one-third lived alone. Did social isolation—and its accompanying manifestations of apathy, depression, and fatigue—play a role in why so many with disease manifestations did not regularly seek medical help? Our results showed that being married was associated with lower odds of natural death compared with those who were single.

### Drug-related deaths

In the current study, whites had 2.22 times higher odds of drug death than Blacks. Findings that drug-related deaths are more prevalent among whites are in keeping with recent literature. For instance, a study of mortality rates in the U.S. between 1999 and 2013 found an increase in mortality among middle-aged white men and women, a change that essentially reversed mortality rates [31]. The trends were that death rates decreased over time for Black and Hispanic groups while rates for whites increased over time, especially for those with less education [7, 31–32]. In another study, results also noted that whites are increasingly dying from opioids [30]. In addition, the author noted that areas where drug-related deaths occurred corresponded with communities in which residents had lower levels of educational attainment [7, 37].

In efforts to address public health concerns of the current substance abuse epidemic, there is a need for additional information on drug-related death certificates. The medical examiner’s setting in which this study occurred routinely provides detailed drug information within the cause of death section versus a blanket statement of drug overdose. Simply stating “drug overdose” was an omission noted in a previously published study [44]. Specifically, the author indicated that death certificates are not sufficient to address upstream initiatives. For example, the current study found that opioids alone accounted for a small percentage of drug-related deaths. In actuality, many of the drug-related deaths involving opioids occurred in combination with other drug substances, especially benzodiazepines [45–46]. Whereas opioids alone accounted for less than 8% of deaths, opioids in combination with other substances accounted for 92.5% deaths.

### Years of potential life lost

The average years of potential life lost due to premature drug deaths was highest for females, i.e., 34.5 YPLL. The current study results support other studies indicating that young females are dying at higher rates from opioids [14, 12, 37]. Also, the lowest YPLL average (27.5 years) was for males dying of natural causes. These findings support a study that addressed the growing concern of young men dying prematurely from treatable diseases [47]. Since YPLL calculations are based on life expectancy, and the life expectancy of males at their time of death was almost 5 years less than that of females, it is not unusual to find lower YPLL values for men. Significant differences of means were found between groups. Individuals who died from drug-related causes had higher YPLLs than individuals dying prematurely from natural causes. What was not answered in the study was the individual’s perceived abilities, health literacy, or actual ability to carry out chronic disease self-management interventions. On the other hand, it is likely that some percentage of premature deaths were preventable.

Limitations of this study include the cross-sectional design and the inclusion of deaths only over a one-year period within one county. Further study incorporating similar data in other counties and in subsequent years would allow for inferences about geographic differences and time trends. Furthermore, this sample was limited to deaths in the county that were under the jurisdiction of the medical examiner’s office. This subsample represents the more underserved segment of the population and findings cannot be generalized to all deaths. Another limitation was the use of aggregate data at the census tract level from the American Community Survey, which is known to have relatively high margins of error [48]. For these small areas of analysis only 5-year estimates are available and it is acknowledged that these are relatively noisy data. Nevertheless, this source of error had relatively little impact on our overall results in that these estimates were used only in the geospatial analysis to describe areas in which there were clusters of natural and drug-related deaths.

### Recommendations for future studies

Although the degree of preventability (i.e., being able to diagnose, manage, or treat the disease) was not ascertained in the present study, the question still exists and should be examined in future studies. The CDC has reported that up to 40% of premature deaths are preventable [13] and 2018 State Health Score Cards noted a marked increase in treatable deaths, both nationally and in two-thirds of the states [49]. To this end, the implications for this present study may translate into data driven interventions, policy, and technology innovations to reduce premature deaths at community levels. Further research should include large sample size population-based studies that address relevant questions and identify barriers to access if effective initiatives are to be devised and implemented.

A comparison of the maps depicting areas of premature deaths (Figs 1 and 2) with the MUA map (Fig 3) shows overlap with areas of premature death. Ascertaining the extent of this overlap for both natural and drug-related deaths should be a priority in future population-based studies. Given the similarity of community characteristics among MUAs and areas of premature death, the notion remains that medical resources were limited and that lack of access, combined with community-based aspects of low income and educational levels, were contributing factors to dying prematurely [50].

## Conclusion

This study went beyond typical studies of cause of death and provided detailed information concerning circumstances of death, disease states, and community resources of those dying prematurely. Descriptive and mapped data presented a more comprehensive picture of the disease state, especially for individuals dying prematurely in low income areas with limited medical access. The information gained in the current study should be used to inform public health initiatives for addressing the goal of reducing premature deaths by 40% in 2030, i.e., the UN sustainable development goal for health [51]. The findings have potential to propel additional research on community-based healthcare in areas of vulnerable communities where there is a high risk of dying prematurely. This, in turn, could impact providers of healthcare services, as community-based programs may be necessary to fill identified gaps in service. In particular, community-based interventions specific to patterns of health behaviors or diseases could be implemented within identified high-risk communities.
